# Channels Adopted for Information Seeking during COVID-19: Comparing Social Media with News Media and Interpersonal Communication in Taiwan

**DOI:** 10.3390/ijerph19159321

**Published:** 2022-07-29

**Authors:** Shu-Chu Sarrina Li, Tai-Yee Wu, Huai-Kuai Zeng, Shih-Yu Lo

**Affiliations:** Institute of Communication Studies, National Yang Ming Chiao Tung University, Hsinchu 30010, Taiwan; taiyeewu@nycu.edu.tw (T.-Y.W.); miazeng.aa07g@nctu.edu.tw (H.-K.Z.); shihyulo@nycu.edu.tw (S.-Y.L.)

**Keywords:** niche theory, COVID-19, information seeking, social media, news media, interpersonal communication, gratifications obtained, gratification opportunities

## Abstract

By adopting niche theory, this study compared social media with news media and interpersonal communication regarding their capabilities in satisfying people’s information needs of daily use, surveillance, convenience, and information quality during the outbreak of COVID-19. Two methods were adopted to collect data for this study: the first was to conduct 20 intensive interviews, and the second was to administer an online survey by contracting a professional polling company with a panel of 8.8 million members. The stratified random sampling method was used to acquire a representative sample, from which 1100 valid questionnaires were obtained. The results showed that: (1) Social media were superior to traditional news media in terms of its convenience. However, several new types of online news, such as Yahoo news, were able to compete with social media for convenience. (2) Interpersonal communication did not outperform in satisfying individuals’ needs for the four gratifications. Nevertheless, interpersonal communication plays the role of social support for individuals.

## 1. Introduction

Although incredible advances have been achieved in medicine and public health, modern crises continue to emerge that impact the health of human beings. COVID-19 is one such crisis, and it has disrupted the normal lives of most people in the world for over 20 months [1]. This novel disease differs from previous ones because the virus is being transmitted rapidly, and medical experts thus far have not reached a consensus regarding effective methods to cure this disease. Therefore, many cities are locked down, schools are closed, business employees are required to work from home, and most mass gatherings, such as sports events, are prohibited to prevent this disease from spreading further [2,3].

Health crises are characterized by a high level of uncertainty. In particular, COVID-19 has been regarded as a mysterious disease because, until now, its origin and the actual scope of its outbreak remain unclear. Moreover, since several variants of COVID-19 have emerged, medical experts may fail to accurately estimate the effectiveness of the existing vaccines in protecting people from being affected by the variants of COVID-19. When facing uncertainty, people seek relevant information to reduce this uncertainty [3]. Under such circumstances, individuals need to obtain timely and accurate information to avoid harm [4]. Adopting niche theory, this study compared three types of communication channels—news media, social media, and interpersonal communication—to validate the advantages and disadvantages regarding the three types of media for communicating risk-relevant information to the public. The results of this study provide governments and health organizations with a good understanding of how to utilize different types of media to communicate various types of information to the public.

Niche theory, originating from organizational ecology, proposes that the key for a medium to survive in its environment is its fit with its environment. This theory assumes that a medium’s fit with its environment plays a more critical part than its internal management for this medium’s survival. By assessing the resource utilization patterns of different media, niche theory explains why these media are able to coexist in an environment of limited resources [5,6,7]. The obtained gratifications measure consumers’ degrees of satisfaction with using a particular medium, which is regarded as the most important resource in the media industry because prior studies have demonstrated that consumer satisfaction is the reason that individuals will continue using a given medium [8,9]. Hence, the gratifications obtained by individuals will bring more customer money, customer time, and advertising revenue to this medium. According to Dimmick [5], assessing the levels of gratification obtained among different media allows scholars to understand the capacities of these media regarding their competitive advantages in fulfilling consumer needs.

The objective of this study is to adopt niche theory to compare the capacities of news media, social media, and interpersonal communication to fulfill people’s need for information during the outbreak of COVID-19 in Taiwan. More specifically, this study classifies obtained gratifications for information into different types and compares the three types of media to understand their capacities to fulfill individuals’ need for these different types of information.

## 2. Literature Review and Hypotheses

### 2.1. Key Concepts in Niche Theory

A niche is defined by Dimmick [5] as a medium’s resource utilization pattern, which includes several aspects in that a medium consumes several types of resources. Niche theory has three important concepts. First, niche breadth, or the area of a niche, labels the quantity and degree of resources utilized by a medium. Based on niche breadth, media can be further classified into two types: generalists and specialists. Generalists utilize many types of resources and have a broad niche. Generalists do not use their resources efficiently, but their advantage is to adjust resource usage quickly when the environment shows tremendous changes. In comparison, specialists utilize only a few types of resources and have a narrow niche. Specialists are efficient resource users, but when facing tremendous changes in their environment, it is difficult for them to adjust their use of resources. The second concept is niche overlap, which is the degree to which the same resources are consumed by two media. The competition between two media is intense when the degree of niche overlap between them is high. The third concept—competitive superiority—is the extent to which one medium’s resource utilization pattern is better than that of another medium. If the two media’s niche overlap is high, the competitive displacement phenomenon is likely to occur, resulting in the inferior medium’s resources being partially seized by the superior medium. According to niche theory, consumers’ time and resources are limited; hence, as they use more resources and time on one type of media, such as social media, consumers leave fewer resources and time available for other media. If one type of media is capable of providing superior functions than other types of media, then these media are better positioned to compete with other types of media. The concept of gratification obtained is the same as that of satisfactions that originate from the theory of uses and gratifications (U&G). According to U&G, individuals use mass media to fulfill their social or psychological needs; hence, when a given medium does not allow individuals to satisfy their needs, they will cease using it and look for other media to fulfill their needs [9]. Therefore, gratifications obtained are the reason for people to continue using a medium. Niche theory incorporates this concept—gratifications obtained—into its theoretical framework and assumes that examining the capacities of different media allows scholars to assess the strengths of these media [5].

Gratification opportunities are related to time and space constraints. Niche theory proposes that when a given medium is able to allow individuals to eliminate time or space constraints, this medium holds a better position than other media in terms of providing more opportunities to gratify individuals’ needs [10].

### 2.2. Gratifications Obtained from Communication Media

With the rapid development of the internet, social media have become prevalent in Taiwan. Facebook, LINE, and Instagram are the most popular social media in Taiwan, among which LINE has the highest penetration rate, with 91.3% of Taiwan’s people using this medium, followed by Facebook and Instagram, with penetration rates of 85.7% and 42.38%, respectively, among those who are aged twelve and above [11]. A recent trend for people in Taiwan is to use social media to obtain news. Studies [12,13] indicate that approximately 83% of Taiwan’s people relied on the internet for news, among which 71% used social media as their major channels for news. Furthermore, LINE (49%) and Facebook (47%) were the most popular social media used by people to obtain news. The existing studies identified four gratifications that people obtained from social media to obtain news: socialization, relationship maintenance, surveillance, and entertainment. During the outbreak of COVID-19, this study surmised that most people used social media to obtain pandemic-related information for surveillance.

Professional news media in this study include cable news channels, print newspapers, and electronic newspapers. Lee’s [14] study identified four gratifications obtained from news consumption: opinion gratification, sociability, information, and entertainment. Li [15] examined the existing studies on news consumption and further divided the gratification of information into information for daily use and information for professional use. Moreover, several studies indicated that surveillance was the gratification obtained across different types of news consumption [14,16]. This study conjectured that when people used news media to seek pandemic information during the outbreak, their main motivations were looking for information for daily use and surveillance.

During the COVID-19 pandemic, seeking relevant information is an important way for people to protect themselves. Therefore, timely information, such as the spread of this disease, the location of the confirmed cases, and recommendations from medical experts, is critical information for people who are trying to protect themselves when conducting daily activities. During the outbreak of COVID-19, most people in Taiwan exercised social distancing, reduced face-to-face interactions, and attended no mass gatherings. Interpersonal communication, which includes face-to-face communication and voice communication, was severely restricted during this time [17]. Therefore, social media and professional news media should be superior to interpersonal communication for providing such information for daily use. Furthermore, past studies [18,19] have shown that individuals in Taiwan obtain science and health news mainly from news media because of news media’s expertise with the two types of news. Thus, the authors estimated that news media should be the best channel for individuals to obtain information for daily use, followed by social media, and interpersonal communication would be the poorest channel for obtaining such information.

**Hypothesis** **1a** **(H1a).** *Regarding information for daily use, news media have the highest degrees, followed by social media, and interpersonal communication has the lowest degrees in niche breadth and competitive superiority.*

Another type of critical information for people to obtain is understanding the impacts of COVID-19 on both Taiwan and other countries. This type of information enables individuals to perform surveillance of their environments, which is the expertise of news media. The literature has shown that the most important gratification news media offer to the public is surveillance [14,16]. Moreover, existing studies have discovered that more than 50% of Taiwanese people read news from social media rather than from news media. Many individuals like to read news and share it on social media [13,20]. Therefore, social media platforms have performed some of the news media’s functions. With these findings, we hypothesized that news media would be the superior media in performing the function of surveillance, social media would be the next, and interpersonal communication would be the poorest.

**Hypothesis** **1b** **(H1b).** *Regarding surveillance, news media have the highest degrees, followed by social media, and interpersonal communication has the lowest degrees in niche breadth and competitive superiority.*

COVID-19 is such an unpredictable disease that when it emerged in Taiwan, people were situated in a highly uncertain context, which prompted people to seek and discuss COVID-19-related information on the internet in great volume. Chin et al. [21] investigated the contents of social media and Google search volume in Taiwan during this time and found that the information from news reports transmitted to the public was instantly transformed into relevant discussions in social media and was also positively correlated with Google search volumes. Various types of information from different sources are available to the public, containing a great deal of misinformation. During the COVID-19 pandemic, misinformation is rife, which causes distress for most people because misinformation contains rumors, incorrect protective measures, and false disease information [3,21]. If people cannot differentiate true from false information, it may result in detrimental effects on their health. Therefore, when individuals select a given medium to obtain information, they are likely to be concerned with the quality of information conveyed by this medium.

News media have expertise in news gathering; thus, the news reports should have the highest quality of information. However, a controversial issue in Taiwan is whether Taiwan should be independent from China or united with China. Most news media take either of the two positions on this issue, which often affects their news reports. Most news media are concerned about their political ideology; thus, if a news event is about China, the news reports by news media with different political ideologies will differ greatly from one another. Therefore, most people in Taiwan consider political news reports by news media to often be distorted and biased [15]. This study predicted that most people in Taiwan would not regard news media as reliable and accurate sources of COVID-19 information. Moreover, news reports from social media come from various sources and are often mixed with misinformation [3,21]. Hence, there is great variation in terms of information quality from social media. This study deemed the information from interpersonal communication to have the highest quality because during the outbreak of COVID-19, the government in Taiwan imposed a restrictive rule on face-to-face communication. Thus, most people only contacted important others, such as family members, friends, and a few trusted colleagues. These people were concerned with each other’s health, and thus, when they conveyed information to their important others, they ensured that the information was accurate [3]. With this reasoning, the following hypothesis was developed:

**Hypothesis** **1c** **(H1c).** *For information quality, interpersonal communication has the highest degrees, followed by social media, with news media having the lowest degrees in niche breadth and competitive superiority.*

According to niche theory, gratification opportunities are important resources for media to possess, which enable individuals to eliminate time and space constraints. Therefore, convenience of use is an important gratification opportunity for communication media. News media include electronic newspapers and cable news channels, which offer updated news almost every hour. In addition, when reporting a complicated issue such as COVID-19, news media often organize all information in a logical sequence for the audience to better understand this issue. Hence, this study hypothesized news media to be the best channel for the gratification of convenience. Currently, many individuals in Taiwan use social media for news. Nevertheless, several studies have indicated that the types of news shared or read in social media differ greatly from those in news media, with social media tending to center on just a few types of news [1,2]. Thus, social media would be less ideal than professional news media but better than interpersonal communication regarding comprehensive news reports. With this reasoning, the following hypothesis was developed:

**Hypothesis** **1d** **(H1d).** *Regarding convenience, news media have the highest degrees, followed by social media, with interpersonal communication having the lowest degrees in niche breadth and competitive superiority.*

### 2.3. Competitive Superiority and Niche Overlap

Niche theory assumes that when the niche overlap between two media is high, the medium with a higher competitive superiority score will gradually replace the medium with a lower superiority score. Empirical studies have also verified this phenomenon [22,23]. For example, Ha and Fang [24] used niche theory to compare traditional news with internet news in the U.S. and discovered that under two conditions, replacement behaviors occurred. The first was for news users to perceive traditional news to be inferior to internet news; the second was for news users to perceive the two types of news to be similar. Therefore, the scores of competitive superiority permit scholars to estimate individuals’ displacement behaviors among different media. With this rationale, the following hypothesis was developed:

**Hypothesis** **2** **(H2).** *The competitive superiority degrees of news media, social media, and interpersonal communication will be positively related to the use of news media, social media, and interpersonal communication.*

## 3. Research Methodology

### 3.1. Interviews on Gratifications Obtained from Three Types of Media

This study first conducted intensive interviews to gather a comprehensive list of gratifications obtained. A snowballing method was adopted to recruit ten females and ten males who had used the three types of media to obtain COVID-19 information. This study purposively diversified interviewees’ gender, education levels, ages, and jobs to obtain various responses. The interviewees’ ages ranged from 21 to more than 56 years old. Their education levels also varied widely from having a senior high-school education to having a doctoral education. This study trained five research assistants who were communication graduate students to conduct intensive interviews. Each interview took approximately 45 min and was recorded and transcribed for later analysis. This study employed the method of content analysis to analyze the interview responses [25]. Only those gratifications that were mentioned by at least three interviewees were selected as an item for the online questionnaire to avoid idiosyncratic personal gratifications. With this procedure, this study formulated seventeen items regarding gratifications obtained from using the three types of media.

### 3.2. Questionnaire for the Online Survey

There were four sections in the questionnaire: the first contained six items that asked how frequently the respondents used each of the three media to obtain COVID-19 information; the second section contained 51 items regarding gratifications obtained, with seventeen items for each of the three media; the third section contained ten items that asked the respondents about their use of social media and mass media; and the last section contained four items on their demographics, including sex, education, age, and personal income. The items of the first two sections used a 7-point Likert scale.

### 3.3. Online Survey

This study collected data by using an online survey method with the target population of Taiwanese adults who were at least 20 years old. A professional polling company, ETtoday, which has an online panel of 8.8 million members, was contracted to conduct this survey. A proportionate stratified random sampling was adopted to draw a sample from the 8.8 million potential participants using age, gender, education, residential location in Taiwan, and personal income as strata. This study required that the sample profile be congruent with that of Taiwan’s online population. According to a recent survey [26], the penetration rate of internet use in 2020 was approximately 88% for Taiwan’s overall population, with people aged between 20 and 54 at 96.73%, people aged between 55 and 64 at 79.5%, and people older than 64 at 42.7%. The online survey was administered from 1 to 18 April 2021, and 1100 valid questionnaires were obtained.

The authors’ university’s Research Ethics Committee for Human Subject Protection approved this study’s data collection procedure before data were collected, and the approval number is NCTU-REC-109-122 W.

### 3.4. Factor Analysis on Gratifications Obtained from Three types of Media

This study needed to have the same set of factors regarding the gratifications obtained from the three types of media; thus, the following procedure was adopted. Three sets of exploratory factor analyses (EFAs) were first conducted on the responses to the 17 items regarding gratifications obtained. The results indicated that the four-factor model on news media had the best outcomes in terms of explained variance and factor loadings. Based on this four-factor model of news media, this study conducted three CFAs (confirmatory factor analyses) on news media, social media, and interpersonal communication by using Mplus 7.4 [27]. Three criteria recommended by statisticians were employed to measure the model fit: RMSEA (<0.10), SRMR (<0.10), and CFI (>0.90) [28,29]. After deleting two items with low factor loadings, the results indicated a good model fit, which are summarized in Table 1.

The first factor was information for daily use, which contained four items concerning the number of confirmed cases, how to protect oneself, the location of confirmed cases, and the spread of this disease in other countries. The second factor, surveillance, contained four items concerning the development of vaccines, the impacts on Taiwan and the world, respectively, and the border control in Taiwan and other countries. The third factor, information quality, contained three items concerning misinformation, second-hand information, and too much trivial information. The fourth factor, convenience, contained four items concerning being quick to obtain updated information, obtaining various types of information, information being convenient to collect, and information is easy to read.

### 3.5. Niche Breadth, Niche Overlap, and Competitive Superiority

The scores of niche breadth for the three types of media were calculated by adopting Formula (1), developed by Dimmick [5] (p. 79). A higher score indicates a wider niche breadth. The scores of niche overlap among the three media were calculated by using Dimmick’s Formula (2) [5] (p. 79). A lower score of niche overlap indicates a higher degree of competition between two media.

The competitive superiority scores were computed by adopting Dimmick’s Formula (3) [5] (p. 80), which contains two sub-formulas, the first of which, SUPERIORITY S_(A > B), is used to measure the degree to which medium A is superior to medium B. The second sub-formula, SUPERIORITY S_(B > A), is used to measure the degree to which medium B is superior to medium A. If scholars want to ensure that medium A is superior to medium B, two conditions are necessary; the first is for the score of the first sub-formula to be higher than that of the second sub-formula. The second is for the results of t-tests on the mean of medium A to be significantly higher than that of medium B [5].

Niche Breadth

(1) $$B=\frac{\sum_{n=1}^{N}\left[\frac{\left(\sum_{k=1}^{K}GO_{n}\right)-Kl}{K\left(u-1\right)}\right]}{N}$$

where: (Dimmick, 2003, [5], p. 79).

Niche Overlap

(2) $$O_{i,j}=\frac{\sum_{n=1}^{N}\sqrt{\sum_{k=1}^{K}\frac{{\left(GO_{i}-GO_{j}\right)}^{2}}{K}}}{N}$$

where: (Dimmick, 2003, [5], p. 79).

Competitive Superiority

(3) $$\mathrm{SUPERIORITY}\;S_{i>j}=\frac{\sum_{n=1}^{N}\sum_{k=1}^{K}\left(m{=}_{i>j}\right)}{N}\;\mathrm{SUPERIORITY}\;S_{j>i}=\frac{\sum_{n=1}^{N}\sum_{k=1}^{K}\left(m{=}_{j>i}\right)}{N}$$

where: (Dimmick, 2003, [5], p. 80).

### 3.6. Sample Profile

Among the 1100 respondents, 51% were males. Regarding age, 19.4% of them were between 20 and 29, 22% were between 30 and 39, 22.3% were between 40 and 49, 19.8% were between 50 and 59, and 16.5% were older than 60. Regarding education, 32.7% received senior high-school education, 47.5% received college education, 18.4% received graduate education, and only 1.5% received junior high-school or elementary school education. Compared to Taiwan’s general population, individuals receiving junior high-school or elementary school education and individuals older than 60 were less represented in this sample [30].

## 4. Research Findings

### 4.1. General Statistics for Major Variables

There were 15 major variables in this study and Table 2 summarizes the general statistical data for these variables.

### 4.2. Gratifications Obtained from the Three Types of Media

Four gratifications were identified from the three types of media: information for daily use, surveillance, information quality, and convenience. The scores of niche breadth, niche overlap, and competitive superiority are summarized in Table 3, Table 4 and Table 5.

As demonstrated in Table 3, news media had higher niche breadth scores than social media, which, in turn, had higher scores than interpersonal communication. Regarding competitive superiority, Table 5 demonstrates that news media had significantly higher scores than social media, which then had higher superiority scores than interpersonal communication. Thus, H1a, H1b, and H1d were supported, but H1c was not supported.

According to Dimmick’s Formula (2) [10], a lower niche overlap score indicates a higher degree of competition between two media. Table 4 indicates that for the three gratifications obtained—convenience, surveillance, and information for daily use—social media and news media had the highest degrees of niche overlap. However, social media and interpersonal communication had the highest level of niche overlap for information quality, followed by social media and news media.

The score of competitive superiority computed from Dimmick’s Formula (3) [10] was summed up from all the respondents’ ratings, which was a superiority score for a medium, not for an individual. This study investigated how an individual superiority score was related to his/her displacement behavior. Thus, following the approach by Gaskins and Jerit [23], this study calculated each respondent’s superiority score, and the calculation method was as follows. This study used a respondent’s rating on a given gratification obtained from social media deducted from that respondent’s rating on the same gratification obtained from interpersonal communication to become that respondent’s superiority score on social media vs. interpersonal communication. Four gratifications obtained from the three types of media were identified; thus, there were twelve individual superiority scores. This study conducted twelve regression analyses using the twelve superiority scores as dependent variables, with the use of social media, news media, and interpersonal communication as independent variables, and three demographic variables—gender, age, and education—as control variables to investigate the relationship between individuals’ superiority scores and their replacement behaviors. The data are summarized in Table 6.

As indicated in Table 6, the superiority scores on social media vs. interpersonal communication were positively correlated with the use of social media but negatively correlated with the use of interpersonal communication for the four gratifications. Similar findings were discovered for the relationships between the superiority scores on social media vs. news media and replacement behaviors and the relationships between the superiority scores on interpersonal communication vs. news media and replacement behaviors. Hence, H2 was supported.

## 5. Discussion

### 5.1. Gratifications Obtained from the Three Types of Media

As predicted by H1a, the data showed that news media were considered the best media to obtain information for daily use, social media were the second best, and interpersonal communication was the poorest for obtaining this gratification during the outbreak of COVID-19 in Taiwan. This result is congruent with past studies’ findings [15,18,19], showing that among science, health, and political news, people in Taiwan regarded health news to be the most relevant to individuals’ everyday lives and thus regarded health news as the most useful for daily use. Moreover, health issues are often complex, and professional news media have the best expertise to explicate the related information to the audience. In addition, our qualitative interviews echoed these results, as eleven interviewees indicated that they were concerned about the number of daily confirmed cases and usually relied on news media for this information. Taiwan’s Center for Disease Control has held daily news conferences since the outbreak of COVID-19, which provides detailed information on confirmed cases, effective methods to protect oneself, and changes in lockdown rules.

Furthermore, the respondents cared about the locations of confirmed cases because they did not want to get close to those locations. Eleven interviewees mentioned that they could gain the most recent updates about this disease from social media, but all the information from different sources seems to collapse on the sites at the same time. Hence, information from social media was too disorganized to understand, while the information from news media was reliable and organized. As indicated by the interviewees, interpersonal communication in this gratification facilitated the exchange of information about how others executed those self-protective measures or the current situations in different cities in Taiwan.

Congruent with the prediction of H1b, this study also found that news media were regarded as the best media for users to fulfill the gratification of surveillance, followed by social media and interpersonal communication. These findings demonstrate that news media primarily function to help surveillance of the environment for the public, resonating with prior research [14,31,32]. In particular, surveillance was discovered to be the gratification obtained across different types of news consumption [14]. As the literature [33] indicates that dependence on news media promoted risk perceptions of the H1N1 pandemic, news media in this study may also enable users to be more fully aware of the potential health risks caused by COVID-19.

Social media also performed part of the surveillance function for the public because many people in Taiwan are exposed to news on social media. However, several studies have indicated that individuals share news on social media not only to maintain relationships with friends but also to develop an ideal self-image, and thus, the types of news shared on social media tend to differ greatly from those reported by news media [34,35]. Therefore, this study considered that news shared on social media was not as comprehensive as news media for the purpose of information seeking. Hence, news media were discovered to perform surveillance better than social media.

Our intensive interviews also echoed these findings. Nine interviewees indicated that they wanted to learn more about this disease in other countries. One interviewee stated that her company had business with clients from European countries. Thus, staying informed about the progress of the pandemic helped her estimate the impact of this disease on her company. Another interviewee responded that he was more concerned about vaccine availability and effectiveness. These interviewees indicated that news media have partnerships with many international news agencies to share each other’s news reports and were thus more capable of providing professional and in-depth analyses for these issues. As indicated in our interviews, social media were also able to provide such information, but various types of information were mixed. Therefore, the depth and professionalism of information were inadequate when compared with those of news media.

For information quality, we hypothesized in H1c that interpersonal communication would be considered the best for this gratification. However, we unexpectedly found that news media were regarded as the best media, social media as the second best, and interpersonal communication as the poorest. The possible reasons for these unexpected findings are that people in Taiwan considered information accuracy the most important when facing such a life-threatening disaster. Information from news media has to pass through several gatekeeping stages, which the respondents deemed to be the most reliable and accurate. News media might have biased news reports when handling news related to political ideology, but there are many news media available in Taiwan, which allowed the respondents to compare and detect the accuracy of news reports. However, compared to news media, news on social media comes from a diversity of sources, and their credibility varies. Moreover, much information on social media was second-hand, which often had the problems of inaccuracy and exaggeration [18,19,34,35]. Our qualitative interviews also echoed these possible interpretations. For example, twelve interviewees indicated that compared to social media, information from news media was more accurate. Furthermore, one frequent approach adopted by the interviewees was to compare the news reports of news media with those of media holding different political ideologies [15]. If the news reports from these media provided similar information, then they could ensure the accuracy of the information. Moreover, eleven interviewees indicated that information from social media was difficult to verify. In particular, they regarded LINE as the least trustworthy social media platform in Taiwan because it contained a lot of misinformation. As the interviewees described, Facebook might not spread as much fake news, but the platform was too chaotic to differentiate true from false information.

In addition, the respondents were found to regard the quality of information from interpersonal communication as the lowest among the three types of communication channels. Information quality is closely related to one’s capacity for information gathering. According to the model of risk information seeking and processing [36,37,38], information gathering capacity positively predicts an individual’s information seeking and processing behaviors, which affect his or her decision-making quality. That is, the quality of information exchanged in interpersonal communication varies, depending greatly on individuals’ information gathering capacity. During the outbreak of COVID-19 in Taiwan, misinformation was rife, and thus, instead of exchanging useful information, most respondents might use interpersonal communication mainly to correct inaccurate information received from others [13]. This interpretation was verified by our intensive interviews with several interviewees. For example, one interviewee was worried that her parents would receive misinformation about the coronavirus from their friends because their parents were not good at using the internet. Therefore, these interviewees actively sought out accurate information for their important others. This finding is congruent with those of Tandoc Jr. and Lee’s study [3] showing that young Singaporeans searched for information during the early stages of the COVID-19 outbreak to correct their parents’ false information.

As predicted by H1d, news media were considered the best media for obtaining the gratification of convenience, social media were the second best, and interpersonal communication was the poorest. This finding is congruent with prior research findings [22,39]. The gratification of convenience in this study contained three aspects: updating news quickly, obtaining a variety of news, and being easy to read. Li’s [22] study showed that people in Taiwan considered television news the best media for obtaining information-related news because television news provided comprehensive news topics to the public. In addition, news channels offered many programs in which important topics were intensively explored and discussed by experts to give the public in-depth knowledge on these topics. For updating news and ease of reading, people in Taiwan regarded Yahoo or Google News as the best media to acquire these gratifications. Google and Yahoo do not produce their news but purchase various types of news from Taiwan’s news organizations to attract more people to visit their portal sites, which has become popular media for young people to obtain news [22,39].

Our interviews also reflected these results. For example, ten interviewees stated that both social media and online news were efficient in providing the most updated information. In particular, several interviewees mentioned that Google or Yahoo news was quick to update its news reports. Seven interviewees talked about online news’ ability to offer more comprehensive news reports than social media. For example, one interviewee indicated that when reading Google news about COVID-19 information, she always clicked the links that would connect her to the website of Common Health magazine, which specializes in health issues. She could thus gain more professional knowledge on this disease. Six interviewees mentioned that news media could organize news information and presented it in a sequential and logical manner for people to process. Regarding the role of interpersonal communication, two interviewees indicated that having conversations with others about COVID-19 either face-to-face or by phone enabled them to ask questions or probe further issues until they clarified their doubts, which was not obtainable in news media or social media. These findings were consistent with those of Kramer, Dougherty, and Pierce [40], showing that when employees heard about their airline company being acquired by another company, they actively used various channels to seek further information. Among them, peer communication was regarded as the most valuable. Although little useful information was exchanged in peer communication, it made employees feel that they were not alone and that they were on the same boat.

### 5.2. Relationship between Replacement Behaviors and Niche Overlap 

Consistent with H2, an individual’s superiority scores predicted his or her replacement behaviors. Table 6 indicates that the superiority scores of social media versus interpersonal communication were positively correlated with social media use but negatively correlated with the use of interpersonal communication. Similar results were obtained for the superiority scores of social media versus news media and the superiority scores of interpersonal communication versus news media. The only exception in Table 6 was the superiority score of social media versus interpersonal communication on the gratification of information quality. According to niche theory, the condition for the superiority scores to predict replacement behaviors is that the users of two media must perceive them as similar. Therefore, a possible explanation for the non-significant relationship between the superiority scores and replacement behaviors on information quality is that users regarded both social media and interpersonal communication as being poor media for providing the gratification of information quality. Therefore, even though the respondents considered social media unable to satisfy their need for information quality, they did not consider interpersonal communication as a good substitute for providing this gratification [22,23,24].

## 6. Conclusions

By using niche theory as a theoretical framework, this study compared social media with news media and interpersonal communication regarding their capabilities in satisfying people’s needs for information for daily use, surveillance, convenience, and information quality during the outbreak of COVID-19 in Taiwan. The results support that niche theory was useful in identifying the strengths and weaknesses of the three communication channels through the calculation of niche breadth, niche overlap, and competitive superiority. In addition, integrating the data from the intensive interviews allowed in-depth data analysis on the competitive advantages of the three channels, further corroborating the findings of this study.

The data analysis yielded three conclusions: (1) News media were regarded as the best media, social media were the second best, and interpersonal communication was the poorest for obtaining the four types of gratification during this pandemic. (2) Social media were superior to traditional news media regarding its convenience. Nevertheless, several new types of online news such as Google News were able to compete with social media in the gratification of convenience. Therefore, news media in general were perceived to be better than social media in this gratification. (3) Interpersonal communication did not outperform in satisfying individuals’ needs with regard to the four gratifications. However, interpersonal communication provides individuals with social support, which is important for people not to feel alone when facing such a life-threatening pandemic.

This study is not without limitations, one of which is that it does not separate online news media from traditional news media. In particular, this study found that several new types of news media, such as Yahoo news and Google news, are more similar to social media than to traditional news media. Future studies should be conducted to examine how traditional news media differ from these new types of news media in light of their abilities to gratify people’s needs for various types of information during the period of disasters. Another limitation is that this study did not measure respondents’ psychological variables. Past studies [38,39,40] have shown that information seeking behaviors are closely associated with individuals’ psychological variables, such as personality or lifestyles. In particular, two personality traits—extraversion and neuroticism—were discovered to differ greatly when seeking risk information [41,42]. Highly extraverted individuals are attracted to positive messages, while highly neurotic individuals pay more attention to negative messages. Therefore, studies [42,43] have demonstrated that highly extraverted subjects interpret the same message differently from highly neurotic subjects. Future studies should be conducted by dividing respondents into two types—high level of extraversion and high level of neuroticism—to compare their gratifications obtained from news media, social media, and interpersonal communication to examine the effect of personality on information seeking behaviors. 

## Figures and Tables

**Table 1:** Confirmatory factor analysis for three media.

Gratifications Obtained	IC ^1^	SM ^2^	NM ^3^
**Factor 1: Information for daily use**			
Knowing how many confirmed cases	0.781	0.731	0.713
Knowing the spread of this disease in other countries	0.811	0.837	0.812
Knowing how to protect oneself	0.801	0.766	0.802
Knowing the location of confirmed cases	0.864	0.849	0.835
**Cronbach α**	**0.946**	**0.939**	**0.937**
**Factor 2: Surveillance**			
The development of vaccines	0.832	0.809	0.765
The impacts on Taiwan	0.839	0.834	0.839
The border control in Taiwan and other countries	0.89	0.855	0.826
The economic impacts on the world	0.87	0.865	0.808
**Cronbach α**	**0.96**	**0.959**	**0.944**
**Factor 3: Convenience**			
Quick to obtain the most updated information	0.857	0.854	0.874
Obtain various types of information	0.916	0.903	0.885
Information is easy to read	0.896	0.854	0.825
Convenient to collect information	0.846	0.816	0.788
**Cronbach α**	**0.966**	**0.96**	**0.955**
**Factor 4: Information quality**			
Lots of misinformation (reversed coding)	0.318	0.317	0.385
Lots of second-hand information (reversed coding)	0.78	0.84	0.841
Too much trivial information (reversed coding)	0.665	0.65	0.696
**Cronbach α**	**0.788**	**0.801**	**0.83**
**Goodness of Fit**			
**X^2^**	630.533	436.449	402.997
**df**	84	84	84
**CFI**	0.973	0.981	0.982
**RMSEA**	0.077	0.062	0.059
**SRMR**	0.07	0.061	0.047
**TLI**	0.966	0.977	0.978

^1^ = Interpersonal communication, ^2^ = social media, ^3^ = news media.

**Table 2:** General statistics for major variables.

Variable	Number of Items	Mean	SD	Min	Median	Max
Frequency of IC ^1^	2	4.01	1.82	1.00	4.00	7.00
Frequency of SM ^2^	2	5.45	1.53	1.00	6.00	7.00
Frequency of NM ^3^	2	5.95	1.18	1.00	6.00	7.00
IC_Information for daily use	4	4.24	1.72	1.00	4.25	7.00
IC_Surveillance	4	4.24	1.76	1.00	4.25	7.00
IC_Convenience	4	4.15	1.74	1.00	4.00	7.00
IC_Information Quality	3	3.19	1.35	1.00	3.33	7.00
SM_Information for daily use	4	5.33	1.43	1.00	5.50	7.00
SM_Surveillance	4	5.22	1.50	1.00	5.25	7.00
SM_Convenience	4	5.15	1.48	1.00	5.25	7.00
SM_Information Quality	3	3.27	1.35	1.00	3.33	7.00
NM_Information for daily use	4	5.89	1.08	1.00	6.00	7.00
NM_Surveillance	4	5.87	1.13	1.00	6.00	7.00
NM_Convenience	4	5.78	1.15	1.00	6.00	7.00
NM_Information Quality	3	3.78	1.57	1.00	4.00	7.00

*N* = 1100, ^1^ = interpersonal communication, ^2^ = social media, ^3^ = news media.

**Table 3:** Niche breadth of the four gratifications.

	Interpersonal Communication	Social Media	News Media
Information for daily use	0.5399	0.7211	0.8153
	News Media > Social Media > Interpersonal Comm		
Surveillance	0.5405	0.7026	0.8117
	News Media > Social Media > Interpersonal Comm		
Convenience	0.5249	0.6917	0.7975
	News Media > Social Media > Interpersonal Comm		
Information quality	0.2323	0.2423	0.3064
	News Media > Social Media > Interpersonal Comm		

**Table 4:** Niche overlap among the three types of media.

	Interpersonal/SM	SM/NM	NM/Interpersonal
Information for daily use	1.45	0.93	1.95
Surveillance	1.27	0.94	1.88
Convenience	1.27	0.94	1.88
Information quality	0.94	0.97	1.54

**Table 5:** Competitive superiority for news media, social media, and interpersonal communication.

Dimensions	IC/SM	SM/NM	NM/IC
Information for daily use	IC < SM	SM < NM	NM > IC
	1.26/9.88	1.95/7.30	13.71/1.23
	t = −21.128 ***	t = −14.009 ***	t = 28.256 ***
News Media > Social Media > Interpersonal Communication (*N* = 1100)			
Surveillance	IC < SM	SM < NM	NM > IC
	1.36/8.87	1.45/7.83	13.51/1.17
	t = −18.872 ***	t = −15.530 ***	t = 27.481 ***
News Media > Social Media > Interpersonal Communication (*N* = 1100)			
Convenience	IC < SM	SM < NM	NM > IC
	1.19/9.41	1.66/7.77	13.64/1.13
	t = −20.244 ***	t = −15.298 ***	t = 28.100 ***
News Media > Social Media > Interpersonal Communication (*N* = 1100)			
Information quality	IC < SM	SM < NM	NM > IC
	2.16/2.75	1.15/4.47	5.64/2.29
	t = −2.155 *	t = −12.873 ***	t = 11.049 ***
News Media > Social Media > Interpersonal Communication (*N* = 1100)			

* *p* < 0.05, *** *p* < 0.001.

**Table 6:** Individual superiority scores and replacement behaviors.

	SM/IC (1)	SM/IC (2)	SM/IC (3)	SM/IC (4)	SM/NM (1)	SM/NM (2)	SM/NM (3)	SM/NM (4)	IC/NM (1)	IC/NM (2)	IC/NM (3)	IC/NM (4)
**Demographics**												
Gender	0.065 *	0.044	0.060 *	0.062 *	0.007	−0.015	−0.004	−0.044	−0.052	−0.049	−0.054	−0.075 *
Age	−0.018	0.029	−0.008	−0.009	−0.145 ***	−0.139 ***	−0.165 ***	−0.064 *	−0.084 **	−0.124 ***	−0.111 ***	−0.041
Edu	0.015	0.017	0.037	0.001	−0.093 **	−0.110 ***	−0.097 **	−0.031	−0.077 *	−0.094 **	−0.100 **	−0.024
R	0.069	0.052	0.069	0.063	0.153 ***	0.155 ***	0.171 ***	0.075	0.108 **	0.141 ***	0.137 ***	0.083
Adjusted R^2^	0.002	0.000	0.002	0.001	0.021	0.021	0.026	0.003	0.009	0.017	0.016	0.004
**Information Seeking**												
Social media	0.531 ***	0.470 ***	0.461 ***	0.060	0.718 ***	0.658 ***	0.622 ***	0.275 ***				
Face to face	−0.672 ***	−0.617 ***	−0.591 ***	−0.037					0.661 ***	0.597 ***	0.583 ***	0.165 ***
Mass media					−0.539 ***	−0.506 ***	−0.486 ***	−0.244 ***	−0.434 ***	−0.393 ***	−0.385 ***	−0.164 ***
R	0.711 ***	0.644 ***	0.624 ***	0.086	0.706 ***	0.655 ***	0.628 ***	0.289 ***	0.753 ***	0.688 ***	0.672 ***	0.234 ***
Adjusted R^2^	0.503	0.412	0.387	0.003	0.497	0.426	0.392	0.080	0.565	0.471	0.449	0.050
Increase adjusted R^2^	0.500	0.412	0.385	0.003	0.476	0.405	0.366	0.078	0.555	0.454	0.433	0.048

* = *p* < 0.05, ** = *p* < 0.001, *** = *p* < 0.001. SM/IC (1) = social media ratings on factor 1—interpersonal communication ratings on factor 1. SM/NM (2) = social media ratings on factor 2—news media ratings on factor 2. IC/NM (3) = interpersonal communication ratings on factor 3—news media ratings on factor 3.

## Data Availability

Please contact the corresponding author for the information of data availability.
